# Leveraging phytochemicals: the plant phylogeny predicts sources of novel antibacterial compounds

**DOI:** 10.2144/fsoa-2018-0124

**Published:** 2019-07-25

**Authors:** Malini A Prasad, Christine P Zolnik, Jeanmaire Molina

**Affiliations:** 1 Department of Biology, Long Island University – Brooklyn, 1 University Plaza, Brooklyn, NY 11201, USA

**Keywords:** antibiotic resistance, evolutionary pharmacology, medicinal plants, phytochemicals, plant antibacterial

## Abstract

**Aim:**

The goal of this study was to use phylogenetic evidence to determine plant families with high representation of antibacterial activity and identify potential sources to focus on for antibacterial drug discovery.

**Materials & methods:**

We reconstructed the molecular phylogeny of plant taxa with antibacterial activity and mapped antibacterial mechanisms of action on the phylogeny.

**Results:**

The phylogeny highlighted seven plant families (Combretaceae, Cupressaceae, Fabaceae, Lamiaceae, Lauraceae, Myrtaceae and Zingiberaceae) with disproportionately represented antibacterial activity. Phytochemicals produced were primarily involved in the disruption of the bacterial cell wall/membrane and inhibition of quorum sensing/biofilm production.

**Conclusion:**

The study provides phylogenetic evidence of seven plant families that should be examined as promising leads for novel antibacterial development.

## Lay abstract

Antibiotic resistance is a growing worldwide problem. However, discovery and development of new antibacterial compounds are currently lacking. For this study, we examined published literature to identify plant groups that disproportionately produce antibacterial compounds. Additionally, we examined the evolutionary basis for these chemicals by determining relatedness of antibacterial-producing plants and their common antibacterial mode of action due to shared ancestry. This study has increased our understanding of plants with inherent antibacterial properties and plant families to target in antibiotic drug discovery studies.

The discovery of penicillin in 1928 was a turning point in human history, providing us with an arsenal to combat deadly bacterial infections [1]. Though antibiotics have been proven effective in the treatment of many bacterial infections over the past century, the emergence of new infectious diseases and the evolution of antibiotic resistance remind us of what may be our incessant battle against bacterial infections [2]. The rapid emergence of resistant bacteria has been attributed to overuse and misuse of these medications [2,3] and consequently, different antibiotics are now required to treat these resistant strains. For example, treatment for an infection by *Staphylococcus aureus* has changed from penicillin to methicillin to vancomycin due to increase in resistant strains [4]. The WHO considers this trend of antibiotic resistance as the most concerning medical dilemma facing us today [5].

Although Fleming accidentally discovered penicillin in the 1920s, it was not until 1965 that the precise mechanism of action was determined – the inhibition of bacterial cell wall (CW) synthesis [6]. In addition to inhibition of peptidoglycan synthesis, different classes of antibiotics act by disrupting cell membrane (CM) functions, inhibiting protein and nucleic acid synthesis, inhibiting DNA replication and transcription and interrupting metabolism (e.g., antimetabolites). Antibiotics can also function as inhibitors of efflux pumps (EP), quorum sensing (QS) and biofilm (BF) formation [7]. Consequently, in an evolutionary arms race, bacteria have acquired resistance to antibiotics by evolving mechanisms that reduce drug uptake (via impermeability or EPs), inactivate drugs via enzymatic attack or through modification of specific target sites [1,3,8]. The evolution and spread of antibiotic resistance have been the greatest threat to successful antibiotic treatment, and hence the driving force behind the search for new therapies [1,2,8].

US FDA approvals have steadily decreased over time, from a peak of 29 new systemic antibiotics approved throughout the 1980s, to just eight new antibiotics in the last decade [9]. Antibiotics in current use are derived from a limited number of chemical classes, sourced mostly from bacteria and fungi that were discovered by the 1960s [2,8]. Subsequent antibiotics have been developed by modifying these chemical scaffolds to reduce toxicity, increase the spectrum of activity or improve efficacy when applied with other antibiotics [1]. For example, dalvabancin and oritavancin, which are among the eight newly US FDA approved antibiotics, belong to the same glycopeptide class as vancomycin, a first generation glycopeptide antibiotic approved by the FDA in 1958 [10]. Resistance to one antibiotic often leads to resistance to other antibiotics within the same class. The evolution of antibiotic resistance has thus outpaced the discovery of new antibiotics [1,2,8].

Different strategies can be applied in the search for novel antibacterial agents. These include the identification of new target sites, repurposing drugs that were initially approved for noninfectious diseases, and sourcing of novel antibiotic producers [2]. Plants, rich in a wide variety of secondary metabolites, represent an auspicious resource of novel antibacterials. Being sessile, their best defense against pathogen attack is to evolve a vast array of chemical weapons [1,3]. Yet, interestingly, none of our modern pharmaceutical antibacterial agents have been developed from plants [8,11], albeit plants have been used since antiquity in traditional medicine to fight bacterial infections [3,8,12]. To date, there has been no documentation of bacteria developing resistance to plant antibacterial compounds [1].

Unlike conventional pharmacotherapeutic agents that are designed to act on a single pathogen target, plant-derived drugs may act as ‘herbal shotgun,’ offering multicomponent therapies with broad-spectrum activities [13]. The combined effects of different active constituents in plant extracts may also work synergistically to enhance antimicrobial efficacy [1]. This is illustrated by the interaction between the antimicrobial alkaloid berberine, and multidrug resistance pump inhibitors, 5′-methoxyhydnocarpin (5′-MHC) and pheophorbide A, which are all produced in various *Berberis* spp. (Berberidaceae). 5′-MHC and pheophorbide A strongly potentiate the action of berberine as it accumulates in bacterial cells, effectively immobilizing plant pathogen efflux mechanisms [11]. This could be why *Berberis* spp. have long been used traditionally as antimicrobials [14]. Fabricant and Farnsworth [12] gathered ethnomedical information from around the world and found that 122 plant-derived pure compounds are used as medicinal drugs, including berberine. The compounds from this study came from only 94 species out of at least 370,000 plant species [15], demonstrating the likelihood that an abundance of antibacterial compounds is yet to be discovered from plants.

The microbial cell can be affected by plant secondary metabolites in several different ways. These include the disruption of membrane function and structure, interruption of DNA/RNA synthesis and function, inactivation of proteins, interference with metabolism and the alteration of cytoplasmic constituents [1,3]. These mechanisms mirror basic methods of antibiotic drug action against bacterial cells. In addition, plants also produce compounds that work as inhibitors of bacterial EP preventing extrusion of antibiotics, as well as inhibitors of QS and BFs that facilitate adhesion and protection of the bacterial population. Yet, surprisingly, there is no plant-derived antibiotic [8]. Clinical studies on the antibacterial effects of plants are rare, albeit there are numerous scientific publications extolling their potent antibacterial properties *in vitro* [1,3,12]. Therefore, the goal of this study is to phylogenetically identify plants that have demonstrated evidence of antibacterial properties, which should be prioritized for novel antibiotic drug development. Recent studies have demonstrated the predictive utility of the plant phylogeny in identifying novel drug sources, exemplifying that pharmacological applications may be gleaned from evolutionary patterns underlying ethnobotanical uses [16–20]. The predictive utility of the phylogeny lies in its power to assign information to nodes instead of one species at a time, thus facilitating discovery of evolutionary trait patterns [21]. Reconstructing the phylogeny of plant species that have shown scientific evidence of antibacterial activity along with their antibacterial mechanisms will facilitate identification of pharmacologically important plant clades with evolutionarily conserved antibacterial phytochemistry. This concept of evolutionary pharmacology [22] directs us to these plant groups and their unexplored species informing us of their potential drug actions based on what is experimentally known in closely related species, thereby potentially being a more expeditious antibiotic drug discovery effort than conducting arbitrary pharmacological assays.

## Materials & methods

Plant taxa with published scientific evidence of antibacterial activity were compiled for this study (Supplementary Table 1). The scientific literature database PubMed was searched using the following ‘plant antibacterial activity, phytochemical and antibacterial, antibacterial plants,’ limiting these terms to the title/abstract without publication date restrictions. We understand that in this way, we may have missed plant species that have no published studies on their antibacterial properties, underestimating the plant species that produce antibacterial compounds. However, this study provides a baseline understanding of plant taxa that may be explored for discovery of plant-derived antibacterial agents.

To build the phylogeny, the *rbcL* gene sequence (∼1300 bp) for each plant species was obtained from GenBank following methods in Guzman and Molina [22]. To ensure that the resulting phylogeny would reflect accepted relationships, we used the *rbcL* gene, which has long been used to elucidate plant phylogenetic relationships [23,24]. Congeneric species were represented once on the phylogeny (e.g., *Alpinia* spp. included *Alpinia nigra* and *Alpinia katsumadae*) to account for taxonomic uncertainties that are common in species circumscriptions and to not visually bias the phylogeny toward a certain family with multispecies genera. The *rbcL* sequence of any congeneric species was chosen when the species cited in the article was not represented in GenBank. The *rbcL* sequences of plants with antibacterial properties were aligned using default parameters in MAFFT [25]. The maximum likelihood phylogeny was reconstructed using PhyML applying the GTR substitution model and SH-like branch support [26]. Interactive Tree of Life, version 2.1 [27], an online tool for the display and manipulation of phylogenetic trees was used to map antibacterial mechanisms. The antibacterial mechanisms of each plant species were characterized as follows: inhibits/degrades bacterial CW and/or cell membrane (CW/CM), inhibits protein and/or nucleic acid synthesis (PN), efflux pump inhibitors (EP), inhibits QS and/or BF production (QS/BF). If mode of action was unknown, unknown mechanism was assigned. Multiple modes of action, when applicable, were indicated for each species. Families, with at least 5% of the genera antibacterial and majority sharing a common antibacterial mechanism were considered disproportionately represented on the phylogeny. This number (5%, antibacterial genera/total number of genera in the family) was the average proportion of antibacterial genera among the over-represented families, while the other families had a lower proportion.

## Results

**Figure 1.**
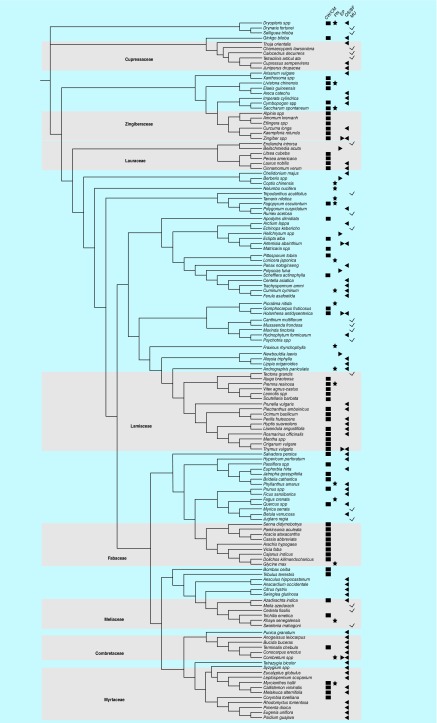
The phylogeny of plant species experimentally shown to possess antibacterial activity. The phylogeny conforms to expected phylogenetic relationships [24]. Plant families that are disproportionately represented in the antibacterial plant phylogeny, with at least three genera, most of which sharing a common mechanism (designated as various symbols to the right, see legend) are labeled and shaded: Combretaceae, Cupressaceae, Myrtaceae as QS/BF, Fabaceae, Lamiaceae, Lauraceae and Zingiberaceae as bacterial CW/CM. At least 5% of their genera are antibacterial (except for Fabaceae, see discussion) and show common modes of action, though for Meliaceae, the MU. Other antibacterial mechanisms of action include PN and EP inhibitors. Overall, there were 61 taxa involved in disruption of CW/CM, 63 in QS/BF inhibition, 19 in PN inhibition, ten with EP effects and 19 with unknown mechanisms. BF: Biofilm; CM: Cell membrane; CW: Cell wall; EP: Efflux pump; MU: Mechanism was unknown; PN: Protein and/or nucleic acid synthesis; QS: Quorum sensing.

**Figure 2.**
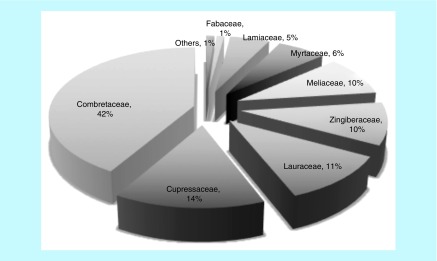
Relative importance of plant families in the antibacterial plant phylogeny after correcting for generic diversity. Some families are inherently diverse, for example, Fabaceae is represented by nine antibacterial genera but has a total of 751 genera so its antibacterial importance is relatively marginal (1%) compared with Combretaceae (42%).

There were 137 antibacterial plant genera (Figure 1; Supplementary Table 1) from 54 families compiled for this study. The phylogeny of these taxa was reconstructed and reflected expected angiosperm taxonomic classification relationships [24]. Antibacterial mechanisms of action were mapped on the phylogeny with 61 taxa involved in disruption of CW/CM, 63 with QS/BF inhibition, 19 in PN inhibition, ten with EP effects and 19 with unknown mechanisms. Seven families were disproportionately represented, with a majority of their genera sharing a common antibacterial mechanism of action possibly due to possession of similar phytochemicals (Figure 1; Table 1). Cupressaceae, Combretaceae and Myrtaceae were predominantly associated with QS/BF inhibition, while Fabaceae, Lamiaceae, Lauraceae and Zingiberaceae demonstrated ability to disrupt the bacterial CW and/or membrane. Flavonoids, tannins and essential oil components were found to be implicated in these antibacterial effects. The percent of each family that was found to contain genera with antibacterial activity was at least 5% (range 5–42%), with the exception of Fabaceae (1%), which is likely due to the high diversity of this family (Figure 2). There was no common antibacterial mechanism identified for Meliaceae, although this was among the families that were over-represented. Plants with phytochemicals that inhibit nucleotide/protein synthesis or EP were sparse throughout the phylogeny.

**Table 1.** Main antibacterial families (at least 5% of genera antibacterial except Fabaceae; see discussion), their known mechanisms of action, and active phytochemicals involved based on the references listed in Supplementary Table 1.

Family	Known mechanism of action	Primary active phytochemicals
Combretaceae	Quorum sensing/biofilm inhibitor	Flavonoids, e.g., catechin, naringenin; ellagic acid, ellagic acid derivatives, ellagitannins
Cupressaceae	Quorum sensing/biofilm inhibitor	Monoterpenes, e.g., limonene, α-pinene, δ-3-carene, α-terpinolene, camphor; sesquiterpenes, e.g., cedrol; polyphenols
Fabaceae	Inhibits cell wall and/or membrane	Phenolics, flavonoids
Lamiaceae	Inhibits cell wall and/or membrane	Terpenes/terpenoids, e.g., 1,8-cineole, pulegone, thymol, carvacrol, linalool, estragole, citral; carnosic acid; dihydroajugapitin; flavonoids, e.g., baicalin
Lauraceae	Inhibits cell wall and/or membrane	Aldehydes (e.g., cinnamaldehyde), aromatic alcohols (eugenol, benzyl alcohol); terpenoids (citral, citronellal, 1,8-cineole); endiandric acid and derivatives
Meliaceae	Mechanism unknown	Triterpenoid, e.g., limonoids
Myrtaceae	Quorum sensing/biofilm inhibitor	Monoterpenes, e.g., 1,8-cineole, α-pinene, α-terpineol; eugenol; flavonoids, e.g., quercetin
Zingiberaceae	Inhibits cell wall and/or membrane	Monoterpenes, e.g., 1,8-cineole, α-pinene, α-terpinene, β-pinene; flavonoids, e.g., isopanduratin A

## Discussion

Seven plant families with similar confamilial mechanisms of antibacterial activity were disproportionately represented on the phylogeny. Combretaceae, Cupressaceae, Lamiaceae, Lauraceae, Myrtaceae, Zingiberaceae, as well as Meliacae, have disproportionate importance – at least 5% of their genera produce antibacterial compounds – though we were unable to find a precise mechanism of action for Meliaceae. Combretaceae, Cupressaceae and Myrtaceae possess phytochemicals that predominantly inhibit bacterial QS/BFs. Lamiaceae, Lauraceae, Zingiberaceae have shown disruptive effects to bacterial CW and/or CM. Only 1% of Fabaceae are antibacterial, which is likely a result of the immense diversity of this family, though members of the family have consistently shown ability to damage CW. It is possible that the over-representation of these families is an artifact of their species diversity; hence, easy accessibility for antibacterial screening experiments, resulting in a greater proportion of published studies on these taxa. While this is possible for Fabaceae, which is the third most speciose angiosperm family [15], Orchidaceae and Asteraceae are also among the most diverse families [15]; yet, they did not appear as pharmacologically important in our study.

The seven antibacterial families have demonstrated inhibitory effects to both Gram-positive and gram-negative bacteria but noted that Gram-positive bacteria were almost always more susceptible. These plant families may indeed be potentially important sources of novel antibiotics with definite mechanisms of action. It is possible that this study underestimated the number of plant families and species that produce antibacterial agents, as it is likely that many have yet to be documented in the scientific literature. Nonetheless, our study provides a baseline understanding of plant taxa that produce antibacterial compounds and warrant further work in exploratory antibacterial screening. We predict that the observed phylogenetic clustering of antibacterial taxa shown here would also be substantiated by a more sophisticated analysis using ‘hot nodes,’ which are nodes that are significantly over-represented in genera with a given property compared with the rest of the tree [28,29]. Phylogenetic clustering of psychoactive plant taxa in certain families observed by Alrashedy and Molina [18] using the same methodology applied here was interestingly comparable with results obtained in an independent study [30] when such a ‘hot nodes’ analysis was applied.

### Cupressaceae, Combretaceae, Myrtaceae – phytochemistry in quorum sensing & biofilm inhibition

QS is a cell-to-cell communication mechanism, regulated by small diffusible signaling molecules called autoinducers, which allow bacteria to respond and adjust their needs in a population density-dependent manner by altering gene expression. Since QS governs many bioprocesses in bacteria, including BF formation, its inhibition may be an ideal target for the development of novel therapeutics [31]. Three plant families have been shown to possess compounds that inhibit QS, and consequently, its regulated processes such as BF formation.

Cupressaceae (cypress family) consists of 29 genera and 149 species of gymnosperms [15]. Phytochemical analyses report that extracts obtained from some cypress species are associated with inhibition of QS/BF. Essential oils from leaves of *Cupressus sempervirens*, primarily composed of the monoterpene α-pinene, create an unfavorable film on substrates that significantly reduces BF surface adhesion [32]. Aqueous extracts from *Juniperus drupacea* are rich in polyphenols that affect BF development and QS-dependent violacein pigment production [33]. Phenolic compounds from extracts of *Thuja orientalis* inhibit bacterial glucosyltransferase, an enzyme that synthesizes extracellular polysaccharides that contribute to BF formation [34]. Though antibacterial mechanism was not described, the monoterpene limonene in *Chamaecyparis lawsonia* [35], as well as essential oil terpenic constituents 3-carene, camphor and cedrol in *Calocedrus decurrens* and *Tetraclinis articulata* [36] were all found to inhibit bacteria.

Mostly phenolic compounds (flavonoids and tannins) have contributed to QS and BF inhibition in the angiosperm dicot families, Combretaceae and Myrtaceae, though terpenoids were also observed to mediate this effect in a few Myrtaceae species. Combretaceae is a relatively small family with 10 genera and 530 species [15]. Extracts containing the flavonoids catechin and naringenin [37] from *Combretum* spp., ellagic acid derivatives from *Terminalia chebula* [38] and from *Anogeissus leiocarpus* [39] have been linked to inhibition of the QS-dependent virulence factors in *Pseudomonas aeruginosa*. One study [40] has noted that extracts of *T. chebula* were even more inhibitory to the gram-negative bacteria *Klebsiella pneumoniae* compared with Gram-positive *S. aureus*. Ellagitannin from *Conocarpus erectus* and *Bucida buceras* extracts have been found to inhibit QS as well [41].

Myrtaceae, a closely related family to Combretaceae, consists of 132 genera and 5,950 species [15]. Extracts obtained from the family show similar anti-QS abilities due to flavonoids and terpenoids. The flavonoid fraction of *Psidium guajava* methanol leaf extract, containing quercetin and quercetin-3-O-arabinoside, was found to inhibit QS-mediated processes including proteolytic and elastolytic activities, swarming motility and BF formation [42]. Flavonoids possessing dihydroxyl moieties in the flavone A-ring backbone, including the flavonoid quercetin, bind to the QS receptors, and significantly reduce their ability to bind to DNA encoding QS-regulated promoters [31]. One study [43] found that QS inhibition from *Syzygium jambos* is due to significant lowering of virulence factors in *P. aeruginosa* by phytol, a terpenoid, and fatty acid components ethyl linoleate and methyl linolenate. Essential oil terpenes such as 1,8-cineole, alpha-pinene, terpineol were also associated with anti-QS effects [41,44]. *Syzygium aromaticum* and *Pimenta dioica* oils, rich in the phenylpropene eugenol, was found to retard BF formation in enterohemorrhagic *E. coli* [45]. Similarly, Polytoxinol^®^, a plant-derived antimicrobial formulation containing extracts from three Myrtaceae species demonstrated superior efficacy in the treatment of chronic osteomyelitis from methicillin-resistant *S. aureus* (MRSA) in an adult male who has been on long term antibiotics prior to Polytoxinol [46]. Polytoxinol affected BF formation even in low concentrations [47].

Some species of Lamiaceae also showed QS/BF inhibition due to their flavonoid and terpenoid content. Baicalin, a flavone glycoside in *Scutellaria* spp., was shown to inhibit the QS receptor TraR [48]. BF formation is consequently affected, though essential oil components (e.g. terpenoids) may directly impact BF, as observed from *C. sempervirens* (Cupressaceae) [32], *Plectranthus amboinicus* (Lamiaceae) [49] and Myrtaceae species [44]. A terpenic skeleton was used as basis to develop an anti-BF synthetic compound [50]. Cupressaceae is unrelated to Combretaceae and Myrtaceae, but the evolution of similar phytochemistry has facilitated similar antibacterial mechanisms, and species in these groups may be tapped as potential sources of novel anti-QS/BF antibiotic compounds.

### Fabaceae, Lamiaceae, Lauraceae, Zingiberaceae – phytochemistry in bacterial cell wall & cell membrane disruption

Gram-positive bacteria are enclosed in a thick cell wall made of peptidoglycan, a network of crosslinked polymers of disaccharide-peptides [7]. This cell wall (CW), vital for bacterial growth and survival, is the target of β-lactam antibiotics such as penicillin and cephalosporin, as well as glycopeptide antibiotics such as vancomycin. The peptidoglycan layer is thinner in gram-negative bacteria and is surrounded by an outer membrane layer of lipopolysaccharides, which could protect the peptidoglycan layer from antibiotic attack [2,7]. The lack of a protective outer membrane makes Gram-positive bacteria more susceptible to the disruption of CW by antibacterial substances. Members of the unrelated plant families Fabaceae, Lamiaceae, Lauraceae and Zingiberaceae have demonstrated phytochemical constituents that affect the integrity of the CW and/or cell membrane (CM), with Gram-positive bacteria more susceptible, though a few species have been reported to permeabilize outer membranes of gram-negative bacteria.

Fabaceae, the bean family, is the third largest flowering plant family, with approximately 751 genera and 19,500 species [15]. A variety of chemically active constituents, such as alkaloids, phenolics, saponins, terpenoids, are produced by members of the family Fabaceae [51], and any of these may be contributing to bacterial CW/CM disruption observed for Fabaceae members represented in the phylogeny. The chromone derivative, acthaside isolated from dried bark of *Acacia ataxacantha* exhibited antibacterial activity against both Gram-positive and Gram-negative microorganisms, though Gram-positive bacteria showed a greater susceptibility overall [52]. In *Dolichos kilimandscharicus*, saponins were found to inhibit Gram-positive bacteria [53]. Gram-positive bacteria also succumbed to the CW-destabilizing effects of terpenoids and flavonoids in extracts of *Cassia abbreviata, Senna didymobotrya*, *Parkinsonia aculeata* [54]. Polyphenolic extracts from *Vicia faba, Cajanus indica* [55], *Arachis hypogea* [56] and other legume species rich in prenylated isoflavonoids and stilbenoids [57] have been shown to possess potent antibacterial activity against Gram-positive bacteria. Though Fabaceae species possess phytochemical diversity that may be synergistically damaging the CW, flavonoids could be one of the primary phytochemicals responsible as they form complexes with soluble proteins on bacterial CWs [58]. The antibacterial effect of flavonoids may be attributed to various causes such as inhibition of bacterial proteins, DNA synthesis, membrane formation and/or energy metabolism [59]. Though the emergence of Fabaceae here as pharmacologically important may be an artifact of its immense species diversity (i.e., easy accessibility), it is noteworthy that phytochemicals within the family are consistently associated with CW disruption in Gram-positive bacteria.

The unrelated monocot family of gingers, Zingiberaceae, consisting of more than 50 genera and approximately 1600 species [15], produce terpenoids and flavonoids [60]. Extracts prepared from members of this family have been implicated in CW and CM disruption in both Gram-positive and Gram-negative bacteria. Though Gram-negative bacteria resisted the effects of methanol extracts of galangal (*Alpinia galangal*), the acetone extract worked better, suggesting nonpolar terpenoid components could be responsible for inhibiting the Gram-negative bacteria [61]. Essential oil terpenes were also observed to lyse the CM in Gram-positive and Gram-negative bacteria from extracts of *Amomum kravanh* [62]. However, only Gram-positive bacteria were affected by methanol extracts obtained from *Etlingera* species [63], and this result was also seen for phenolic extracts in other Zingiberaceae species [64]. The flavonoid, isopanduratin A, damaged the CW of the Gram-positive cariogenic bacteria, *Streptococcus mutans* [65]. From these studies polyphenolics such as flavonoids may be implicated in CW disruption, like in Fabaceae, while terpenoid components, when extracted from Zingiberaceae using appropriate solvents, could additionally inhibit Gram-negative bacteria. Further work is needed to substantiate these observations.

The Lauraceae family consists of approximately 45 genera and 2850 species [15]. Essential oils of *Cinnamomum verum, Laurus nobilis* and *Litsea cubeba* were shown to cause irreversible bacterial membrane damage [66–70]. Active constituents in their essential oils include aldehydes such as cinnamaldehyde, aromatic alcohols (eugenol, benzyl alcohol) and terpenoids (citral, citronellal, 1,8-cineole). *L. cubeba* and *C. verum* essential oils were noted to disrupt the outer membrane of Gram-negative bacteria [69,70]. Unique to the family are phytochemicals known as endiandric acid and derivatives, which have been shown to have potent antibacterial activity [71].

In the unrelated mint family, Lamiaceae, similar aromatic essential oils components were also found to disrupt bacterial CM integrity. The family consists of 241 genera and 7530 species [15]. Monoterpenes 1,8-cineole from *Vitex agnus-castus* [72] and *Rosmarinus officinalis* [73], pulegone from *Mentha* species, as well as thymol and carvacrol from *Thymus vulgaris* and *Origanum vulgare* [74] disrupted CM. Although the mode of action of thymol and carvacrol is not clearly understood, it is mostly believed that the hydroxyl group on these two compounds interacts with the cytoplasmic membrane, changes its permeability, leading to disruption and leakage of cellular contents [75]. Essential oil components are also shown to cause structural alterations on the outer membrane disrupting it through the release of lipopolysaccharide (i.e., membrane permeabilizers). Carvacrol and thymol have revealed potential to interact with the outer membrane with consequent bactericidal activity [76]. Outer membrane disruption was also reported for *Lavandula angustifolia* due to its terpenoid components linalyl anthranilate and linalool [77]. Other terpenoids such as estragole/methyl chavicol and citral in *Ocimum basilicum* [78], carnosic acid in *R. officinalis* [73] dihydroajugapitin and acetylharpagide from bugle weed, *Ajuga bracteosa* [79] were also involved in interruption of CWs and/or CMs.

As discussed, flavonoids and terpenoids from Fabaceae, Zingiberaceae, Lamiaceae and Lauraceae may be implicated in bacterial CW/CM disruption with Gram-positive bacteria noted to be more susceptible, though a few studies have shown that terpenoids may permeabilize outer membranes of Gram-negative bacteria. Other closely related species of these families may be investigated for similar effects.

### Antibacterial phylogenetic patterns & synergistic phytochemical interactions

Although there were observed phylogenetic patterns in antibacterial mechanisms, similar phytochemicals were implicated in these different mechanisms. CW/CM disruption and anti-QS/BF were the common mechanisms of action tested in the literature evaluated for this study. This focus on these specific mechanisms may potentially be reflecting a bias by the authors of these studies, testing only one of the many possible mechanisms of action that can exist. For example, certain plant species were only tested for CW/CM disruption, but not inhibition of EP, though this may also be a potential mode of action. This may be why there were only fewer instances of inhibition of EP and nucleic acid synthesis as these activities were not routinely assayed. Mechanisms may have also been mistaken for another. Multiple mechanisms of action that are observed in some plants may be due to cellular structures being affected in a cascade type of action. For example, lipophilic extracts can accumulate on CW surfaces, and/or pass through the cytoplasmic membrane. Increased permeability can cause bacterial CW lysis leading to a cascade of disruptive events such as ion leakage, ATP depletion and interruption of the proton pump [80].

Synergy among components may also result in multiple antibacterial mechanisms and could confound associations between phytochemistry and mechanism of action, which may be why we found overlapping phytochemistry for different mechanisms. Synergistic interactions among extract constituents make it more difficult for pathogens to develop antibiotic resistance. For instance, 5′-MHC, an EP inhibitor produced in *Berberis* spp. possess no antimicrobial activity by itself, but it disables plant pathogen resistance mechanisms against berberine, an antimicrobial alkaloid in the plant [11]. A petroleum ether extraction of essential oils in the mint species, *Mentha piperita*, containing primarily menthol, was found to be antibacterial, but an ethyl acetate extract, which included polar phenolic components (e.g., flavonoids), was found to be even more inhibitory, demonstrating synergistic antibacterial effects among components of the extract [81]. When used in combination with antibiotics, natural products may potentiate antibiotic effects. A 2015 study [82] found that *O. basilicum* (Lamiaceae) essential oil coupled with either imipenem or ciprofloxacin demonstrated synergism against clinical strains of *P. aeruginosa* and *S. aureus* restoring β-lactam antibiotic efficacy. This was also observed in ethyl acetate extracts of *Curcuma longa* (Zingiberaceae) lowering minimum inhibitory concentrations of ampicillin and oxacillin against MRSA. Essential oils from *C. verum* (Lauraceae) and *L. angustifolia* (Lamiaceae) are capable of reversing *E. coli* J53 R1 resistance to piperacillin through modifying outer membrane permeability [70,77]. Therefore, in addition to their inherent antibacterial natural products that may be explored for drug development, these plant extracts may also be used in conjunction with pharmaceutical antibiotics to enhance the latter’s effects.

Among the families that are disproportionately represented in this study, some common bioactive secondary metabolites are discernible. Though phylogenetically unrelated, these plant families possessed similar antibacterial phytochemistry, suggesting convergent evolution and/or differential gene regulation of a common metabolic pathway [83]. In a broad sense, much of the observed antibacterial activities can be linked to the presence of essential oil terpenes and terpenoids, and phenolic compounds. The monoterpene oxide 1,8-cineole/eucalyptol was a major constituent in the essential oils of species in the unrelated families of Zingiberaceae, Lamiaceae, Myrtaceae and Lauraceae species, and may therefore play a role in bacterial CM disruption, as well as QS/BF inhibition. Terpene isomers α-pinene and/or β-pinene were present in the essential oils of Cupressaceae, Lamiaceae, Myrtaceae and Zingiberaceae. Flavonoids were present in the alcohol extracts of all seven families but were especially prominent in Combretaceae, Fabaceae, Lamiaceae, Myrtaceae and Zingiberaceae. Flavonoids influence the downregulation of QS virulence factor expression. Moreover, many Meliaceae genera have been shown here to have antibacterial effects, possibly due to nortriterpenoids known as limonoids [84], though a common mechanism is still unclear. Though an apparent relationship between mechanism of action and phytochemistry was lacking, there is no doubt that certain plant families are disproportionately important as sources of antibacterial compounds and exemplifies the utility of the phylogeny in drug discovery [16,17,19] and the applications of evolutionary pharmacology [22]. Our study directs focus on natural products derived from certain plant clades and to test them for these mechanisms of action first to avoid time-consuming hit-and-miss antibacterial assays. As a case in point, the plant-derived antimicrobial formulation, Polytoxinol, primarily sourced from Myrtaceae essential oils, was more efficacious than pharmaceutical antibiotics in treating MRSA due to its BF-inhibiting effects [47]. This mechanism was also predicted by the phylogeny for Myrtaceae. Thus, development of structurally similar drugs with antibacterial phytochemical moieties (e.g., monoterpenes) as templates could usher in more efficient and less toxic antibacterials [3]. Standardized extracts derived from species of these antibacterial plant families may also be administered in conjunction with conventional antibiotics, sensitizing bacteria and enhancing susceptibility to the antibiotic. For instance, extracts from Lamiaceae/Lauraceae species, which have membrane-permeabilizing effects, could potentiate the effect of antibiotics with intracellular targets such as tetracyclines. Therefore, the ability of plant compounds to ‘repurpose’ conventional antibiotics [1] could mitigate evolution of antibiotic resistance. Thus, this study demonstrates a novel, yet underappreciated, avenue for antibacterial drug discovery. Further investigation and development of plant-derived antibacterial agents may finally give us the upper hand in the evolutionary arms race against antibiotic resistance in bacterial pathogens.

## Conclusion & future perspective

Due to the increase in antibiotic resistance, coupled with the limited research and funding devoted to identifying new antibiotics, there is a crucial need to identify novel compounds to combat bacterial infections [85]. Although some plants produce phytochemicals with antibacterial mechanisms of action, extensive investigation into identification of plant taxa of interest, as well as clinical studies and approval are currently lacking.

We provide a baseline understanding of the evolutionary basis of known phytochemicals with antibacterial activity, identifying specific plant taxa to focus drug discovery efforts. Phylogenetic evidence across 137 plant genera within 54 families demonstrated that seven families are disproportionally represented based on their antibacterial effects in *in vitro* experiments. Although it is likely that this study has underestimated the number of plant taxa and lineages with antibacterial activity due to limitations in experimental studies, our results provide a foundational starting point for exploratory antibiotic screening and specific mechanisms of action to focus on. Natural products derived from these plant groups have potential pharmacological value and could also be used as templates for the development of novel antibacterial agents and/or as auxiliary agents to conventional antibiotics to mitigate the problem of antibiotic resistance.

As the emergence of antibiotic resistant bacteria increases [86], the resulting public health and economic burden necessitate discovery and testing of new potential sources to fight this growing global threat. However, most public health initiatives focus on management strategies such as improving diagnosis, optimizing known therapeutic protocols, tracking prescriptions and preventing transmission. New pharmaceutical discovery and development programs continue to focus on traditional antibiotics derived from microorganisms [87]. Phytochemicals represent a valuable, yet underappreciated and understudied, source of novel antibacterial compounds and future studies that expand on this work could provide us with unique compounds for antibacterial drug discovery and innovative strategies for combating antibiotic-resistant bacterial infections.

**Summary points**

**The need for novel antibiotics**

- The evolution of antibiotic resistance is one of the greatest public health challenges of our time, driving the need to develop new antibacterial compounds.
- Plants have long been used traditionally to fight bacterial infections but no US FDA approved pharmaceutical antibacterials have been developed from plant natural products.

**The plant phylogeny as a tool for drug discovery**

- We reconstructed the phylogeny of plant species with antibacterial activity and phylogenetically mapped their antibacterial mechanisms of action to determine if closely related species possess the same bioactivity due to evolutionarily conserved phytochemistry.
- Phylogenetic results highlighted seven families (Combretaceae, Cupressaceae, Fabaceae, Lamiaceae, Lauraceae, Myrtaceae and Zingiberaceae) containing phytochemicals primarily involved in disruption of bacterial cell wall/membrane and inhibition of quorum sensing/biofilms.

**New plant-derived antibiotics**

- Antibacterial effects were mainly mediated by flavonoids and terpenes, which may serve as precursors or templates for antibiotic development and/or as auxiliary agents to conventional antibiotics to mitigate antibiotic resistance.

**Conclusion**

- The phylogeny has identified plant groups that disproportionately produce antibacterial compounds compared with other plants. Natural products from these plants can potentially be exploited as sources of novel antibacterials with definite mechanisms of action.
