# Supplementary material for: Leveraging phytochemicals: the plant phylogeny predicts sources of novel antibacterial compounds
Source: Future Sci OA. 2019 Jul 25;5(7):FSO407. doi: 10.2144/fsoa-2018-0124 (PMC6695524; doi:10.2144/fsoa-2018-0124)
Supplement: Supplementary file 1 [file fsoa-05-407-s1.docx]

Supplementary Table S1

**Antibacterial plant taxa used in this study**. Antibacterial plant taxa, their mechanisms of action, and corresponding GenBank numbers are tabulated. The *rbcL* sequence of another congeneric species was used when the exact species cited in the article was not available in GenBank.

| **Species** | **Family** | **Reference** | **Antibacterial mechanism** | **Accession number** |
| --- | --- | --- | --- | --- |
| *Andrographis paniculata* | Acanthaceae | [1-3] | PN, QS/BF | JQ933217 |
| *Anacardium occidentale* | Anacardiaceae | [4,5] | QS/BF | AY462008 |
| *Centella asiatica* | Apiaceae | [6,7] | QS/BF | D44559 |
| *Cuminum cyminum* | Apiaceae | [8] | PN, QS/BF | JQ933290 |
| *Ferula asafoetida* | Apiaceae | [9] | QS/BF | KY794561 |
| *Trachyspermum ammi* | Apiaceae | [10] | QS/BF | KP974260 |
| *Gomphocarpus fruticosus* | Apocynaceae | [11] | CW/CM | HM850038 |
| *Holarrhena antidysenterica* | Apocynaceae | [2,12] | CW/CM, EP, QS/BF | AJ002884 |
| *Picralima nitida* | Apocynaceae | [13] | PN | X91766 |
| *Arisarum vulgare* | Araceae | [14] | QS/BF | HM849791 |
| *Xanthosoma spp.* | Araceae | [15] | CW/CM | AJ007543 |
| *Panax notoginseng* | Araliaceae | [16] | QS/BF | JF950030 |
| *Polyscias fulva* | Araliaceae | [17] | EP | JX572870 |
| *Schefflera actinophylla* | Araliaceae | [11] | CW/CM | KT748629 |
| *Areca catechu* | Arecaceae | [16] | QS/BF | GQ436757 |
| *Elaeis guineensis* | Arecaceae | [18] | CW/CM | AJ404830 |
| *Livistona chinensis* | Arecaceae | [19] | CW/CM, PN | AJ404757 |
| *Arctium lappa* | Asteraceae | [20] | QS/BF | KM360645 |
| *Artemisia absinthium* | Asteraceae | [21,22] | QS/BF, EP | KX581996 |
| *Eclipta alba* | Asteraceae | [23] | CW/CM | AY215108 |
| *Helichrysum spp.* | Asteraceae | [24,25] | EP | GU817766 |
| *Matricaria spp.* | Asteraceae | [26] | CW/CM | MG223597 |
| *Berberis spp.* | Berberidaceae | [27,28] | EP | KX344612 |
| *Betula verrucosa* | Betulaceae | [29] | QS/BF | KM360670 |
| *Newbouldia laevis* | Bignoniaceae | [17] | EP | KJ735962 |
| *Lonicera japonica* | Caprifoliaceae | [30] | PN | HM850134 |
| *Anogeissus leiocarpus* | Combretaceae | [31] | QS/BF | AF425709 |
| *Bucida buceras* | Combretaceae | [32] | QS/BF | FJ381807 |
| *Combretum spp.* | Combretaceae | [33-35] | PN, EP, QS/BF | EU338166 |
| *Terminalia chebula* | Combretaceae | [36,37] | CW/CM, QS/BF | FJ3818122 |
| *Conocarpus erectus* | Combretaceae | [32] | QS/BF | AF281477 |
| *Echinops kebericho* | Compositae | [38] | MU | KC589850 |
| *Calocedrus decurrens* | Cupressaceae | [39] | MU | KX832621 |
| *Chamaecyparis lawsoniana* | Cupressaceae | [40] | MU | L12570 |
| *Cupressus sempervirens* | Cupressaceae | [41] | QS/BF | L12571 |
| *Juniperus drupacea* | Cupressaceae | [42] | QS/BF | HM024301 |
| *Tetraclinis articulata* | Cupressaceae | [39] | MU | HM024346 |
| *Thuja orientalis* | Cupressaceae | [43] | QS/BF | L12578.2 |
| *Dryopteris spp.* | Dryopteridaceae | [44-46] | CW/CM, PN, QS/BF | KC896537 |
| *Bridelia cathartica* | Euphorbiaceae | [11] | CW/CM | JF265314 |
| *Euphorbia hirta* | Euphorbiaceae | [47] | QS/BF | MF135387 |
| *Jatropha gossypiifolia* | Euphorbiaceae | [48] | CW/CM | KP898356 |
| *Acacia ataxacantha* | Fabaceae | [49] | CW/CM | JF265242 |
| *Glycine max* | Fabaceae | [50] | PN | LT576825 |
| *Arachis hypogaea* | Fabaceae | [51] | CW/CM | KX257487 |
| *Cajanus indicus* | Fabaceae | [52] | CW/CM | KU729879 |
| *Cassia abbreviata* | Fabaceae | [11] | CW/CM | JF265329 |
| *Dolichos kilimandscharicus* | Fabaceae | [53] | CW/CM | AM235006 |
| *Parkinsonia aculeata* | Fabaceae | [11] | CW/CM | EF101290 |
| *Senna didymobotrya* | Fabaceae | [11] | CW/CM | Z70154 |
| *Vicia faba* | Fabaceae | [52] | CW/CM | KF042344 |
| *Quercus spp.* | Fagaceae | [32,54-56] | CW/CM, QS/BF | MF044885 |
| *Fagus crenata* | Fagaceae | [57] | PN | MF044982 |
| *Ginkgo biloba* | Ginkgoaceae | [58,59] | CW/CM, QS/BF | DQ069500 |
| *Hypericum perforatum* | Hypericaceae | [60] | QS/BF | AF206779 |
| *Apodytes dimidiata* | Icacinaceae | [61] | CW/CM | AJ428895 |
| *Juglans regia* | Juglandaceae | [62] | MU | F167464 |
| *Ajuga bracteosa* | Lamiaceae | [63] | CW/CM | KP172044 |
| *Hyptis suaveolens* | Lamiaceae | [64] | QS/BF | JQ591782 |
| *Lavandula angustifolia* | Lamiaceae | [65] | CW/CM, QS/BF | KT948988 |
| *Leonotis spp.* | Lamiaceae | [11] | CW/CM | KR736498 |
| *Mentha spp.* | Lamiaceae | [66] | CW/CM | KP172042 |
| *Ocimum basilicum* | Lamiaceae | [67] | CW/CM | KY623639 |
| *Origanum vulgare* | Lamiaceae | [68] | CW/CM | HM850213 |
| *Perilla frutescens* | Lamiaceae | [69,70] | CW/CM, QS/BF | FJ513160 |
| *Plectranthus amboinicus* | Lamiaceae | [71,72] | CW/CM, QS/BF | KX783974 |
| *Premna resinosa* | Lamiaceae | [73] | CW/CM, PN | KF496428 |
| *Prunella vulgaris* | Lamiaceae | [16] | QS/BF | HM850288 |
| *Rosmarinus officinalis* | Lamiaceae | [74] | CW/CM, QS/BF | Z37435 |
| *Scutellaria barbata* | Lamiaceae | [75] | CW/CM | MF521633 |
| *Tectona grandis* | Lamiaceae | [76] | MU | AJ001765 |
| *Thymus vulgaris* | Lamiaceae | [77-79] | CW/CM, EP, QS/BF | Z37472 |
| *Vitex agnus-castus* | Lamiaceae | [80] | CW/CM | U78716 |
| *Beilschmiedia acuta* | Lauraceae | [81] | EP | KC628468 |
| *Cinnamomum verum* | Lauraceae | [82] | CW/CM, QS/BF | KY635878 |
| *Endiandra introrsa* | Lauraceae | [83] | MU | KT588614 |
| *Laurus nobilis* | Lauraceae | [84] | CW/CM, QS/BF | HM850111 |
| *Litsea cubeba* | Lauraceae | [85] | CW/CM | AY337734 |
| *Persea americana* | Lauraceae | [86] | CW/CM | JF966611 |
| *Tripodanthus acutifolius* | Loranthaceae | [86] | MU | EU544475 |
| *Punica granatum* | Lythraceae | [16] | QS/BF | JQ730672 |
| *Bombax ceiba* | Malvaceae | [87] | CW/CM | AY328181 |
| *Tetrazygia bicolor* | Melastomataceae | [56] | QS/BF | KX397981 |
| *Azadirachta indica* | Meliaceae | [88-90] | CW/CM, QS/BF | AJ402917 |
| *Cedrela fissilis* | Meliaceae | [91] | MU | NC_037251 |
| *Khaya senegalensis* | Meliaceae | [35] | PN | AB925375 |
| *Melia azedarach* | Meliaceae | [92] | MU | MG946851 |
| *Swietenia mahogani* | Meliaceae | [93] | MU | FN599465 |
| *Trichilia emetica* | Meliaceae | [11] | CW/CM | U39082 |
| *Ficus sansibarica* | Moraceae | [94] | QS/BF | JX572600 |
| *Myrica serrata* | Myricaceae | [95] | MU | KM360891 |
| *Callistemon viminalis* | Myrtaceae | [56,96] | CW/CM, QS/BF | JX856666 |
| *Corymbia torelliana* | Myrtaceae | [97] | CW/CM | KF496869 |
| *Eucalyptus globulus* | Myrtaceae | [64,98] | QS/BF | HM849985 |
| *Eugenia uniflora* | Myrtaceae | [99] | QS/BF | AF294255 |
| *Leptospermum scoparium* | Myrtaceae | [100] | QS/BF | HM850121 |
| *Melaleuca alternifolia* | Myrtaceae | [101,102] | CW/CM, QS/BF | AM235658 |
| *Myrcianthes hallii* | Myrtaceae | [103] | CW/CM, PN | AB586448 |
| *Pimenta dioica* | Myrtaceae | [104] | QS/BF | KY085891 |
| *Psidium guajava* | Myrtaceae | [105] | QS/BF | KX527097 |
| *Rhodomyrtus tomentosa* | Myrtaceae | [106] | QS/BF | KX527095 |
| *Syzygium spp* | Myrtaceae | [104,107] | QS/BF | JX856783 |
| *Nelumbo nucifera* | Nelumbonaceae | [108] | PN | KT119346 |
| *Fraxinus rhynchophylla* | Oleaceae | [109] | PN | KP088609 |
| *Chelidonium majus* | Papaveraceae | [110] | QS/BF | KM360713 |
| *Passiflora spp.* | Passifloraceae | [15,111] | CW/CM | DQ123353 |
| *Phyllanthus amarus* | Phyllanthaceae | [112] | PN, QS/BF | MF135411 |
| *Pittosporum tobira* | Pittosporaceae | [11] | CW/CM | HM850261 |
| *Cymbopogon spp.* | Poaceae | [64,113] | CW/CM, QS/BF | KJ740992 |
| *Imperata cylindrica* | Poaceae | [16] | QS/BF | KC164313 |
| *Saccharum spontaneum* | Poaceae | [114] | CW/CM, PN | LN849912 |
| *Fagopyrum esculentum* | Polygonaceae | [115] | CW/CM, PN | AB000309 |
| *Polygonum cuspidatum* | Polygonaceae | [116] | QS/BF | AB019031 |
| *Rumex acetosa* | Polygonaceae | [117,118] | MU | AY395559 |
| *Drynaria fortunei* | Polypodiaceae | [119] | MU | EU128499 |
| *Phymatopteris triloba* | Polypodiaceae | [120] | MU | AY459174 |
| *Coptis chinensis* | Ranunculaceae | [121] | PN | FJ449856 |
| *Prunus spp.* | Rosaceae | [16,122] | CW/CM, QS/BF | AF411489 |
| *Canthium multiflorum* | Rubiaceae | [123] | MU | AM117212 |
| *Hydnophytum formicarum* | Rubiaceae | [124] | QS/BF | X83645 |
| *Morinda tinctoria* | Rubiaceae | [125] | MU | AJ318448 |
| *Mussaenda frondosa* | Rubiaceae | [125] | MU | AJ318447 |
| *Psychotria spp.* | Rubiaceae | [125] | MU | AJ002180 |
| *Citrus hystrix* | Rutaceae | [126,127] | QS/BF | AB505939 |
| *Swinglea glutinosa* | Rutaceae | [64] | QS/BF | AB505960 |
| *Salvadora persica* | Salvadoraceae | [128,129] | CW/CM, QS/BF | KU757320 |
| *Aesculus hippocastanum* | Sapindaceae | [110] | QS/BF | KM360616 |
| *Tamarix nilotica* | Tamaricaceae | [35] | PN | KX298993 |
| *Aloysia triphylla* | Verbenaceae | [64,130] | QS/BF | KY085903 |
| *Lippia origanoides* | Verbenaceae | [64] | QS/BF | KR736512 |
| *Alpinia spp* | Zingiberaceae | [131,132] | CW/CM | KF304130 |
| *Amomum kravanh* | Zingiberaceae | [133] | CW/CM | MF991963 |
| *Curcuma longa* | Zingiberaceae | [134,135] | CW/CM, QS/BF | KX608614 |
| *Etlingera spp* | Zingiberaceae | [136] | CW/CM | [MH603418](https://www.ncbi.nlm.nih.gov/nucleotide/MH603418.1?report=genbank&log$=nucltop&blast_rank=1&RID=0JXMK6XT015) |
| *Kaempferia rotunda* | Zingiberaceae | [137] | CW/CM | KF304172 |
| *Zingiber spp.* | Zingiberaceae | [138,139] | CW/CM, EP, QS/BF | KJ871883 |
| *Tribulus terrestris* | Zygophyllaceae | [140] | CW/CM | DQ267165 |

References for Table S1:

1. Shaikh RU, Dawane AA, Pawar RP, Gond DS, Meshram RJ, Gacche RN. Inhibition of Helicobacter pylori and Its Associate Urease by Labdane Diterpenoids Isolated from Andrographis paniculata. *Phytother Res.* 30(3), 412-417 (2016).

2. Tanwar A, Chawla R, Chakotiya AS *et al.* Effect of Holarrhena antidysentrica (Ha) and Andrographis paniculata (Ap) on the biofilm formation and cell membrane integrity of opportunistic pathogen Salmonella typhimurium. *Microb Pathog* 101, 76-82 (2016).

3. Banerjee M, Parai D, Chattopadhyay S, Mukherjee SK. Andrographolide: antibacterial activity against common bacteria of human health concern and possible mechanism of action. *Folia Microbiol* 62(3), 237-244 (2017).

4. Anand G, Ravinanthan M, Basaviah R, Shetty AV. In vitro antimicrobial and cytotoxic effects of Anacardium occidentale and Mangifera indica in oral care. *J Pharm Bioallied Sci.*7(1), 69-74 (2015).

5. Menezes K, Pereira JV, Nóbrega D, Freitas A, Pereira M, Pereira A. Antimicrobial and anti-adherent in vitro activity of tannins isolated from Anacardium occidentale Linn. (Cashew) on dental biolfilm bacteria. *Pesqui Bras Odontopediatria Clin Integr* 14(3), 191-198 (2014).

6. Vasavi HS, Arun AB, Rekha PD. Anti-quorum sensing activity of flavonoid-rich fraction from Centella asiatica L. against Pseudomonas aeruginosa PAO1. *J Microbiol Immunol Infect* 49(1), 8-15 (2016).

7. Jose D, Lekshmi N, Goel AK, Kumar RA, Thomas S. Development of a Novel Herbal Formulation To Inhibit Biofilm Formation in Toxigenic Vibrio cholerae. *J Food Prot.*1933-1940 (2017).

8. Naveed R, Hussain I, Tawab A *et al.* Antimicrobial activity of the bioactive components of essential oils from Pakistani spices against Salmonella and other multi-drug resistant bacteria. *BMC Complement Altern Med* 13, 265 (2013).

9. Sepahi E, Tarighi S, Ahmadi FS, Bagheri A. Inhibition of quorum sensing in Pseudomonas aeruginosa by two herbal essential oils from Apiaceae family. *J Microbiol.* 53(2), 176-180 (2015).

10. Khan R, Zakir M, Khanam Z, Shakil S, Khan AU. Novel compound from Trachyspermum ammi (Ajowan caraway) seeds with antibiofilm and antiadherence activities against Streptococcus mutans: a potential chemotherapeutic agent against dental caries. *J. Appl. Microbiol.* 109(6), 2151-2159 (2010).

11. Madureira AM, Ramalhete C, Mulhovo S, Duarte A, Ferreira MJ. Antibacterial activity of some African medicinal plants used traditionally against infectious diseases. *Pharm Biol* 50(4), 481-489 (2012).

12. Siriyong T, Srimanote P, Chusri S *et al.* Conessine as a novel inhibitor of multidrug efflux pump systems in Pseudomonas aeruginosa. *BMC Complement Altern Med* 17(1), 405 (2017).

13. Kouitcheu LB, Tamesse JL, Kouam J. The anti-shigellosis activity of the methanol extract of Picralima nitida on Shigella dysenteriae type I induced diarrhoea in rats. *BMC Complement Altern Med* 13, 211 (2013).

14. Majik MS, Naik D, Bhat C, Tilve S, Tilvi S, D'Souza L. Synthesis of (R)-norbgugaine and its potential as quorum sensing inhibitor against Pseudomonas aeruginosa. *Bioorg. Med. Chem. Lett.* 23(8), 2353-2356 (2013).

15. Dzotam JK, Touani FK, Kuete V. Antibacterial and antibiotic-modifying activities of three food plants (Xanthosoma mafaffa Lam., Moringa oleifera (L.) Schott and Passiflora edulis Sims) against multidrug-resistant (MDR) Gram-negative bacteria. *BMC Complement Altern Med* 16, 9 (2016).

16. Koh KH, Tham FY. Screening of traditional Chinese medicinal plants for quorum-sensing inhibitors activity. *J Microbiol Immunol Infect* 44(2), 144-148 (2011).

17. Tankeo SB, Tane P, Kuete V. In vitro antibacterial and antibiotic-potentiation activities of the methanol extracts from Beilschmiedia acuta, Clausena anisata, Newbouldia laevis and Polyscias fulva against multidrug-resistant Gram-negative bacteria. *BMC Complement Altern Med* 15, 412 (2015).

18. Vijayarathna S, Zakaria Z, Chen Y, Latha LY, Kanwar JR, Sasidharan S. The Antimicrobial efficacy of Elaeis guineensis: characterization, in vitro and in vivo studies. *Molecules* 17(5), 4860-4877 (2012).

19. Kaur G, Singh RP. Antibacterial and membrane damaging activity of Livistona chinensis fruit extract. *Food Chem. Toxicol.* 46(7), 2429-2434 (2008).

20. Rajasekharan SK, Ramesh S, Bakkiyaraj D, Elangomathavan R, Kamalanathan C. Burdock root extracts limit quorum-sensing-controlled phenotypes and biofilm architecture in major urinary tract pathogens. *Urolithiasis* 43(1), 29-40 (2015).

21. Fiamegos YC, Kastritis PL, Exarchou V *et al.* Antimicrobial and efflux pump inhibitory activity of caffeoylquinic acids from Artemisia absinthium against gram-positive pathogenic bacteria. *PLOS ONE* 6(4), e18127 (2011).

22. Elmanama A, Al-Reefi M. Antimicrobial, anti-biofilm, anti- quorum sensing, antifungal and synergistic effects of some medicinal plants extracts. *IUGNES* 25(2), 198-207 (2017).

23. Ray A, Bharali P, Konwar BK. Mode of antibacterial activity of Eclalbasaponin isolated from Eclipta alba. *Appl Biochem Biotechnol* 171(8), 2003-2019 (2013).

24. Aelenei P, Miron A, Trifan A, Bujor A, Gille E, Aprotosoaie AC. Essential Oils and Their Components as Modulators of Antibiotic Activity against Gram-Negative Bacteria. *Medicines (Basel)* 3(3) (2016).

25. Lorenzi V, Muselli A, Bernardini AF *et al.* Geraniol restores antibiotic activities against multidrug-resistant isolates from gram-negative species. *Antimicrob Agents Chemother* 53(5), 2209-2211 (2009).

26. Abad MJ, Bedoya LM, Bermejo P. Chapter 14 - Essential Oils from the Asteraceae Family Active against Multidrug-Resistant Bacteria. In: *Fighting Multidrug Resistance with Herbal Extracts, Essential Oils and Their Components.* Rai MK, Kon KV (Eds.), Academic Press, San Diego, 205-221 (2013).

27. Stermitz FR, Lorenz P, Tawara JN, Zenewicz LA, Lewis K. Synergy in a medicinal plant: antimicrobial action of berberine potentiated by 5'-methoxyhydnocarpin, a multidrug pump inhibitor. *Proc. Natl. Acad. Sci. U.S.A* 97(4), 1433-1437 (2000).

28. Rao M, Padyana S, Dipin K, Kumar S, Nayak B, Varela M. Antimicrobial compounds of plant origin as efflux pump inhibitors: new avenues for controlling multidrug resistant pathogens. *J Antimicrob Agents* 4(1) (2018).

29. Tolmacheva AA, Rogozhin EA, Deryabin DG. Antibacterial and quorum sensing regulatory activities of some traditional Eastern-European medicinal plants. *Acta Pharm* 64(2), 173-186 (2014).

30. Kim SJ, Yoon SJ, Kim YM *et al.* HS-23, Lonicera japonica extract, attenuates septic injury by suppressing toll-like receptor 4 signaling. *J Ethnopharmacol*. 155(1), 256-266 (2014).

31. Ouedraogo V, Kiendrebeogo M. Methanol Extract from Anogeissus leiocarpus (DC) Guill. et Perr. (Combretaceae) Stem Bark Quenches the Quorum Sensing of Pseudomonas aeruginosa PAO1. *Medicines (Basel)* 3(4), 26 (2016).

32. Adonizio AL, Downum K, Bennett BC, Mathee K. Anti-quorum sensing activity of medicinal plants in southern Florida. *J Ethnopharmacol*. 105(3), 427-435 (2006).

33. Nyambuya T, Mautsa R, Mukanganyama S. Alkaloid extracts from Combretum zeyheri inhibit the growth of Mycobacterium smegmatis. *BMC Complement Altern Med* 17(1), 124 (2017).

34. Mtisi B, S SS, Mombeshora M, Mukanganyama S. Inhibition of Biofilm Formation in Candida albicans and Candida krusei by Combretum zeyheri Leaf Extracts. *J Bacteriol Mycol.* 5(3), 1-8 (2018).

35. Mohieldin EAM, Muddathir AM, Mitsunaga T. Inhibitory activities of selected Sudanese medicinal plants on Porphyromonas gingivalis and matrix metalloproteinase-9 and isolation of bioactive compounds from Combretum hartmannianum (Schweinf) bark. *BMC Complement Altern Med* 17(1), 224 (2017).

36. Li K, Lin Y, Li B *et al.* Antibacterial constituents of Fructus Chebulae Immaturus and their mechanisms of action. *BMC Complement Altern Med* 16, 183 (2016).

37. Sarabhai S, Sharma P, Capalash N. Ellagic acid derivatives from Terminalia chebula Retz. downregulate the expression of quorum sensing genes to attenuate Pseudomonas aeruginosa PAO1 virulence. *PLOS ONE* 8(1), e53441 (2013).

38. Ameya G, Gure A, Dessalegn E. Antimicrobial Activity Of Echinops Kebericho Against Human Pathogenic Bacteria And Fungi. *Afr J Tradit Complement Altern Med* 13(6), 199-203 (2016).

39. Ibrahim TA, El-Hela AA, El-Hefnawy HM, Al-Taweel AM, Perveen S. Chemical Composition and Antimicrobial Activities of Essential Oils of Some Coniferous Plants Cultivated in Egypt. *Iran J Pharm Res.* 16(1), 328-337 (2017).

40. Pala-Paul J, Usano-Alemany J, Granda E, Soria AC. Antifungal and antibacterial activity of the essential oil of Chamaecyparis lawsoniana from Spain. *Nat Prod Commun* 7(10), 1383-1386 (2012).

41. Selim SA, Adam ME, Hassan SM, Albalawi AR. Chemical composition, antimicrobial and antibiofilm activity of the essential oil and methanol extract of the Mediterranean cypress (Cupressus sempervirens L.). *BMC Complement Altern Med* 14, 179 (2014).

42. Marino A, Bellinghieri V, Nostro A *et al.* In vitro effect of branch extracts of Juniperus species from Turkey on Staphylococcus aureus biofilm. *FEMS Immunol. Med. Microbiol.* 59(3), 470-476 (2010).

43. Choi HA, Cheong DE, Lim HD *et al.* Antimicrobial and Anti-Biofilm Activities of the Methanol Extracts of Medicinal Plants against Dental Pathogens Streptococcus mutans and Candida albicans. *J Microbiol Biotechnol* 27(7), 1242-1248 (2017).

44. Tsou LK, Lara-Tejero M, RoseFigura J *et al.* Antibacterial Flavonoids from Medicinal Plants Covalently Inactivate Type III Protein Secretion Substrates. *J Am Chem Soc* 138(7), 2209-2218 (2016).

45. Jung JE, Pandit S, Jeon JG. Identification of linoleic acid, a main component of the n-hexane fraction from Dryopteris crassirhizoma, as an anti-Streptococcus mutans biofilm agent. *Biofouling* 30(7), 789-798 (2014).

46. Gao C, Guo N, Li N *et al.* Investigation of antibacterial activity of aspidin BB against Propionibacterium acnes. *Arch. Dermatol. Res.* 308(2), 79-86 (2016).

47. Perumal S, Mahmud R. Chemical analysis, inhibition of biofilm formation and biofilm eradication potential of Euphorbia hirta L. against clinical isolates and standard strains. *BMC Complement Altern Med* 13, 346 (2013).

48. Okoh S, Iweriebor B, Oko O, Nwodo U, Okoh A. Antibacterial and antioxidant properties of the leaves and stem essential oils of Jatropha gossypifolia L. *BioMed Res. Int.* 2016, 1-9 (2016).

49. Amoussa AM, Bourjot M, Lagnika L, Vonthron-Senecheau C, Sanni A. Acthaside: a new chromone derivative from Acacia ataxacantha and its biological activities. *BMC Complement Altern Med* 16(1), 506 (2016).

50. Wang Q, Wang H, Xie M. Antibacterial mechanism of soybean isoflavone on Staphylococcus aureus. *Arch Microbiol* 192(11), 893-898 (2010).

51. Tamura T, Ozawa M, Tanaka N, Arai S, Mura K. Bacillus cereus Response to a Proanthocyanidin Trimer, a Transcriptional and Functional Analysis. *Curr Microbiol* 73(1), 115-123 (2016).

52. Chanda S, Dudhatra S, Kaneria M. Antioxidative and antibacterial effects of seeds and fruit rind of nutraceutical plants belonging to the Fabaceae family. *Food Funct.* 1(3), 308-315 (2010).

53. Shava C, Mutsaka P, Moyo B, Sithole S, Chitemerere T, Mukanganyama S. Antibacterial and Anticancer Properties of Dolichos kilimandscharicus (Fabaceae). *JBAPN* 6(2), 112-135 (2016).

54. Chusri S, Phatthalung PN, Voravuthikunchai SP. Anti-biofilm activity of Quercus infectoria G. Olivier against methicillin-resistant Staphylococcus aureus. *Lett. Appl. Microbiol.* 54(6), 511-517 (2012).

55. Chusri S, Voravuthikunchai SP. Damage of staphylococcal cytoplasmic membrane by Quercus infectoria G. Olivier and its components. *Lett. Appl. Microbiol.* 52(6), 565-572 (2011).

56. Adonizio A, Kong KF, Mathee K. Inhibition of quorum sensing-controlled virulence factor production in Pseudomonas aeruginosa by South Florida plant extracts. *Antimicrob Agents Chemother* 52(1), 198-203 (2008).

57. Dubreuil JD. Antibacterial and antidiarrheal activities of plant products against enterotoxinogenic Escherichia coli. *Toxins (Basel)* 5(11), 2009-2041 (2013).

58. He J, Wang S, Wu T, Cao Y, Xu X, Zhou X. Effects of ginkgoneolic acid on the growth, acidogenicity, adherence, and biofilm of Streptococcus mutans in vitro. *Folia Microbiol* 58(2), 147-153 (2013).

59. Bajpai V, Sharma A, Baek K. Antibacterial mode of action of Ginkgo biloba leaf essential oil: effect on morphology and membrane permeability. *Bangladesh J Pharmacol.* 10(2), 337-350 (2015).

60. Lyles JT, Kim A, Nelson K *et al.* The Chemical and Antibacterial Evaluation of St. John's Wort Oil Macerates Used in Kosovar Traditional Medicine. *Front Microbiol.* 8, 1639 (2017).

61. Komape NP, Bagla VP, Kabongo-Kayoka P, Masoko P. Anti-mycobacteria potential and synergistic effects of combined crude extracts of selected medicinal plants used by Bapedi traditional healers to treat tuberculosis related symptoms in Limpopo Province, South Africa. *BMC Complement Altern Med* 17(1), 128 (2017).

62. Rather MA, Dar BA, Dar MY *et al.* Chemical composition, antioxidant and antibacterial activities of the leaf essential oil of Juglans regia L. and its constituents. *Phytomedicine* 19(13), 1185-1190 (2012).

63. Ganaie HA, Ali MN, Ganai BA, Meraj M, Ahmad M. Antibacterial activity of 14, 15-dihydroajugapitin and 8-o-acetylharpagide isolated from Ajuga bracteosa Wall ex. Benth against human pathogenic bacteria. *Microb Pathog* 103, 114-118 (2017).

64. Cervantes-Ceballos L, Caballero-Gallardo K, Olivero-Verbel J. Repellent and Anti-quorum Sensing Activity of Six Aromatic Plants Occurring in Colombia. *Nat Prod Commun* 10(10), 1753-1757 (2015).

65. Yap PS, Krishnan T, Yiap BC, Hu CP, Chan KG, Lim SH. Membrane disruption and anti-quorum sensing effects of synergistic interaction between Lavandula angustifolia (lavender oil) in combination with antibiotic against plasmid-conferred multi-drug-resistant Escherichia coli. *J Appl Microbiol Biochem.* 116(5), 1119-1128 (2014).

66. Oumzil H, Ghoulami S, Rhajaoui M *et al.* Antibacterial and antifungal activity of essential oils of Mentha suaveolens. *Phytother Res* 16(8), 727-731 (2002).

67. Shirazi MT, Gholami H, Kavoosi G, Rowshan V, Tafsiry A. Chemical composition, antioxidant, antimicrobial and cytotoxic activities of Tagetes minuta and Ocimum basilicum essential oils. *Food Sci Nutr* 2(2), 146-155 (2014).

68. Fournomiti M, Kimbaris A, Mantzourani I *et al.* Antimicrobial activity of essential oils of cultivated oregano (Origanum vulgare), sage (Salvia officinalis), and thyme (Thymus vulgaris) against clinical isolates of Escherichia coli, Klebsiella oxytoca, and Klebsiella pneumoniae. *Microb Ecol Health Dis.* 26, 23289 (2015).

69. Sun S, Li H, Zhou W, Liu A, Zhu H. Bacterial Quorum Sensing Inhibition Activity of the Traditional Chinese Herbs, Ficus carica L. and Perilla frutescens. *Chemotherapy* 60(5-6), 379-383 (2014).

70. Witzke S, Duelund L, Kongsted J, Petersen M, Mouritsen OG, Khandelia H. Inclusion of terpenoid plant extracts in lipid bilayers investigated by molecular dynamics simulations. *J Phys Chem B.* 114(48), 15825-15831 (2010).

71. Gupta SK, Negi PS. Antibacterial Activity of Indian Borage (Plectranthus amboinicus Benth) Leaf Extracts in Food Systems andAgainst Natural Microflora in Chicken Meat. *Food Technol. Biotechnol.* 54(1), 90-102 (2016).

72. Vasconcelos S, Melo HM, Cavalcante TTA *et al.* Plectranthus amboinicus essential oil and carvacrol bioactive against planktonic and biofilm of oxacillin- and vancomycin-resistant Staphylococcus aureus. *BMC Complement Altern Med* 17(1), 462 (2017).

73. Njeru SN, Obonyo MA, Nyambati SO, Ngari SM. Antimicrobial and cytotoxicity properties of the crude extracts and fractions of Premna resinosa (Hochst.) Schauer (Compositae): Kenyan traditional medicinal plant. *BMC Complement Altern Med* 15, 295 (2015).

74. Moreno S, Galván E, Vázquez N, G GF, PAC PG. Antibacterial efficacy of Rosmarinus officinalis phytochemicals against nosocomial multidrug-resistant bacteria grown in planktonic culture and biofilm. In: *The Battle Against Microbial Pathogens: Basic Science, Technological Advances and Educational Programs.* Méndez-Vilas, A (Ed.), Formatex Research Centre, Badajoz, Spain, 3-8 (2015).

75. Tang Q-L, Kang A-R, Lu C-X. Phytochemical analysis, antibacterial activity and mode of action of the methanolic extract of Scutellaria barbata against various clinically important bacterial pathogens. *Int J Pharmacol.*, 12, 116-125 (2016).

76. Bitchagno GTM, Sama Fonkeng L, Kopa TK *et al.* Antibacterial activity of ethanolic extract and compounds from fruits of Tectona grandis (Verbenaceae). *BMC Complement Altern Med* 15(1), 265 (2015).

77. Fani M, Kohanteb J. In Vitro Antimicrobial Activity of Thymus vulgaris Essential Oil Against Major Oral Pathogens. *Evid Based Complementary Altern Med.* 22(4), 660-666 (2017).

78. Tankeo SB, Lacmata ST, Noumedem JA, Dzoyem JP, Kuiate JR, Kuete V. Antibacterial and antibiotic-potentiation activities of some Cameroonian food plants against multi-drug resistant gram-negative bacteria. *Chin J Integr Med.* 20(7), 546-554 (2014).

79. Mohsenipour Z, Hassanshahian M. The Effects of Allium sativum Extracts on Biofilm Formation and Activities of Six Pathogenic Bacteria. *Jundishapur J Microbiol* 8(8), e18971 (2015).

80. Stojković D, Soković M, Glamočlija J *et al.* Chemical composition and antimicrobial activity of Vitex agnus-castus L. fruits and leaves essential oils. *Food Chem.* 128(4), 1017-1022 (2011).

81. Chouna JR, Nkeng-Efouet PA, Lenta BN *et al.* Antibacterial endiandric acid derivatives from Beilschmiedia anacardioides. *Phytochemistry* 70(5), 684-688 (2009).

82. Yap PS, Krishnan T, Chan KG, Lim SH. Antibacterial Mode of Action of Cinnamomum verum Bark Essential Oil, Alone and in Combination with Piperacillin, Against a Multi-Drug-Resistant Escherichia coli Strain. *J Microbiol Biotechnol* 25(8), 1299-1306 (2015).

83. Lenta BN, Chouna JR, Nkeng-Efouet PA, Sewald N. Endiandric Acid Derivatives and Other Constituents of Plants from the Genera Beilschmiedia and Endiandra (Lauraceae). *Biomolecules* 5(2), 910-942 (2015).

84. Merghni A, Marzouki H, Hentati H, Aouni M, Mastouri M. Antibacterial and antibiofilm activities of Laurus nobilis L. essential oil against Staphylococcus aureus strains associated with oral infections. *Pathol. Biol.* (2015).

85. Li WR, Shi QS, Liang Q, Xie XB, Huang XM, Chen YB. Antibacterial activity and kinetics of Litsea cubeba oil on Escherichia coli. *PLOS ONE* 9(11), e110983 (2014).

86. Guzman-Rodriguez JJ, Lopez-Gomez R, Suarez-Rodriguez LM *et al.* Antibacterial activity of defensin PaDef from avocado fruit (Persea americana var. drymifolia) expressed in endothelial cells against Escherichia coli and Staphylococcus aureus. *Biomed Res Int* 2013, 986273 (2013).

87. Masood Ur R, Akhtar N, Mustafa R. Antibacterial And Antioxidant Potential Of Stem Bark Extract Of Bombax Ceiba Collected Locally From South Punjab Area Of Pakistan. *Afr J Tradit Complement Altern Med* 14(2), 9-15 (2017).

88. Mahfuzul Hoque MD, Bari ML, Inatsu Y, Juneja VK, Kawamoto S. Antibacterial activity of guava (Psidium guajava L.) and Neem (Azadirachta indica A. Juss.) extracts against foodborne pathogens and spoilage bacteria. *Foodborne Pathog Dis.* 4(4), 481-488 (2007).

89. Del Serrone P, Toniolo C, Nicoletti M. Neem (Azadirachta indica A. Juss) Oil to Tackle Enteropathogenic Escherichia coli. *Biomed Res Int* 2015, 343610 (2015).

90. Alzohairy M. Therapeutics role of Azadirachta indica (Neem) and their active constituents in diseases prevention and treatment. *Evid Based Complement Alternat Med* 1-11 (2016).

91. Lago JHG, de Ávila Jr P, de Aquino EM *et al.* Volatile oils from leaves and stem barks of Cedrela ﬁssilis (Meliaceae): chemical composition and antibacterial activities. *Flavour Fragr J.* 19(5), 448-451 (2004).

92. Khan AV, Ahmed QU, Mir MR, Shukla I, Khan AA. Antibacterial efficacy of the seed extracts of Melia azedarach against some hospital isolated human pathogenic bacterial strains. *Asian Pac J Trop Biomed* 1(6), 452-455 (2011).

93. Rahman AK, Chowdhury AK, Ali HA *et al.* Antibacterial activity of two limonoids from Swietenia mahagoni against multiple-drug-resistant (MDR) bacterial strains. *J Nat Med* 63(1), 41-45 (2009).

94. Awolola GV, Koorbanally NA, Chenia H, Shode FO, Baijnath H. Antibacterial and anti-biofilm activity of flavonoids and triterpenes isolated from the extracts of Ficus sansibarica Warb. subsp. sansibarica (Moraceae) extracts. *Afr J Tradit Complement Altern Med* 11(3), 124-131 (2014).

95. Gafner S, Wolfender JL, Mavi S, Hostettmann K. Antifungal and antibacterial chalcones from Myrica serrata. *Planta Med* 62(1), 67-69 (1996).

96. Salem MZ, Ali HM, El-Shanhorey NA, Abdel-Megeed A. Evaluation of extracts and essential oil from Callistemon viminalis leaves: antibacterial and antioxidant activities, total phenolic and flavonoid contents. *Asian Pac J Trop Dis* 6(10), 785-791 (2013).

97. Nobakht M, Trueman SJ, Wallace HM, Brooks PR, Streeter KJ, Katouli M. Antibacterial Properties of Flavonoids from Kino of the Eucalypt Tree, Corymbia torelliana. *Plants (Basel)* 6(3) (2017).

98. Al-Shuneigat J, Cox SD, Markham JL. Effects of a topical essential oil-containing formulation on biofilm-forming coagulase-negative staphylococci. *Lett. Appl. Microbiol.* 41(1), 52-55 (2005).

99. Rodrigues AC, Zola FG, Avila Oliveira B *et al.* Quorum Quenching and Microbial Control through Phenolic Extract of Eugenia Uniflora Fruits. *J Food Sci.* 81(10), M2538-m2544 (2016).

100. Truchado P, López-Gálvez F, Gil MI, Tomás-Barberán FA, Allende A. Quorum sensing inhibitory and antimicrobial activities of honeys and the relationship with individual phenolics. *Food Chem.* 115(4), 1337-1344 (2009).

101. Kavanaugh NL, Ribbeck K. Selected antimicrobial essential oils eradicate Pseudomonas spp. and Staphylococcus aureus biofilms. *Appl Environ Microbiol* 78(11), 4057-4061 (2012).

102. Carson CF, Mee BJ, Riley TV. Mechanism of action of Melaleuca alternifolia (tea tree) oil on Staphylococcus aureus determined by time-kill, lysis, leakage, and salt tolerance assays and electron microscopy. *Antimicrob Agents Chemother.* 46(6), 1914-1920 (2002).

103. Chavez Carvajal P, Coppo E, Di Lorenzo A *et al.* Chemical Characterization and in Vitro Antibacterial Activity of Myrcianthes hallii (O. Berg) McVaugh (Myrtaceae), a Traditional Plant Growing in Ecuador. *Materials (Basel)* 9(6) (2016).

104. Vasavi HS, Arun AB, Rekha PD. Inhibition of quorum sensing in Chromobacterium violaceum by Syzygium cumini L. and Pimenta dioica L. *Asian Pac J Trop Biomed* 3(12), 954-959 (2013).

105. Vasavi HS, Arun AB, Rekha PD. Anti-quorum sensing activity of Psidium guajava L. flavonoids against Chromobacterium violaceum and Pseudomonas aeruginosa PAO1. *Microbiol Immunol.* 58(5), 286-293 (2014).

106. Limsuwan S, Homlaead S, Watcharakul S *et al.* Inhibition of microbial adhesion to plastic surface and human buccal epithelial cells by Rhodomyrtus tomentosa leaf extract. *Arch Oral Biol*  59(12), 1256-1265 (2014).

107. Musthafa KS, Sianglum W, Saising J, Lethongkam S, Voravuthikunchai SP. Evaluation of phytochemicals from medicinal plants of Myrtaceae family on virulence factor production by Pseudomonas aeruginosa. *APMIS* 125(5), 482-490 (2017).

108. Huh M, Kim H. Antibacterial effect on leaf-extract from Nelumbo nucifera against oral microorganism. *J Dent Hyg* 14(1), 117-221 (2014).

109. Wang H, Zou D, Xie K, Xie M. Antibacterial mechanism of fraxetin against Staphylococcus aureus. *Mol Med Rep* 10(5), 2341-2345 (2014).

110. Artini M, Papa R, Barbato G *et al.* Bacterial biofilm formation inhibitory activity revealed for plant derived natural compounds. *Bioorg. Med. Chem. Lett.* 20(2), 920-926 (2012).

111. Ramaiya S, Bujang J, Zakaria M. Assessment of total phenolic, antioxidant, and antibacterial activities of Passiflora species. *ScientificWorldJournal*, 2014, 1-10 (2014).

112. Priya K, Yin WF, Chan KG. Anti-quorum sensing activity of the traditional Chinese herb, Phyllanthus amarus. *Sensors (Basel)* 13(11), 14558-14569 (2013).

113. Nguefack J, Budde BB, Jakobsen M. Five essential oils from aromatic plants of Cameroon: their antibacterial activity and ability to permeabilize the cytoplasmic membrane of Listeria innocua examined by flow cytometry. *Lett. Appl. Microbiol.* 39(5), 395-400 (2004).

114. Hussaqin M, Ullah R, Raza S *et al.* Assessment of antibacterial potential of Saccharum spontaneum Linn. (family: Poaceae), against different pathogenic microbes- an in vitro study. *J Pharm Alt Med.* 3(3), 36-41 (2014).

115. Cabarkapa I, Sedej I, Sakač M, Saric L, Plavšić D. Antimicrobial activity of buckwheat (Fagopyrum esculentum Moench) hulls extract. *Food and Feed Research* *35*, 159-163 (2008).

116. Coenye T, Brackman G, Rigole P *et al.* Eradication of Propionibacterium acnes biofilms by plant extracts and putative identification of icariin, resveratrol and salidroside as active compounds. *Phytomedicine* 19(5), 409-412 (2012).

117. Schmuch J, Beckert S, Brandt S *et al.* Extract from Rumex acetosa L. for prophylaxis of periodontitis: inhibition of bacterial in vitro adhesion and of gingipains of Porphyromonas gingivalis by epicatechin-3-O-(4beta-->8)-epicatechin-3-O-gallate (procyanidin-B2-Di-gallate). *PLOS ONE* 10(3), e0120130 (2015).

118. Kota S, Dumpala P, Anantha RK, Verma MK, Kandepu S. Evaluation of therapeutic potential of the silver/silver chloride nanoparticles synthesized with the aqueous leaf extract of Rumex acetosa. *Sci Rep.* 7(1), 11566 (2017).

119. Jung E. Antimicrobial activity of extract and fractions from Drynaria fortunei against oral bacteria. *J Bacteriol Virol.* 37(2), 61-68 (2007).

120. Chai TT, Elamparuthi S, Yong AL, Quah Y, Ong HC, Wong FC. Antibacterial, anti-glucosidase, and antioxidant activities of selected highland ferns of Malaysia. *Bot Stud.* 54(1), 55 (2013).

121. Si L, Li P, Liu X, Luo L. Chinese herb medicine against Sortase A catalyzed transformations, a key role in gram-positive bacterial infection progress. *J. Enzyme Inhib. Med. Chem* 31(sup1), 184-196 (2016).

122. Aires A, Dias C, Carvalho R, Saavedra MJ. Analysis of glycosylated flavonoids extracted from sweet-cherry stems, as antibacterial agents against pathogenic Escherichia coli isolates. *Acta Biochim Pol* 64(2), 265-271 (2017).

123. Kouam SF, Ngouonpe AW, Bullach A, Lamshoft M, Kuigoua GM, Spiteller M. Monoterpenes with antibacterial activities from a Cameroonian medicinal plant Canthium Multiflorum (Rubiaceae). *Fitoterapia* 91, 199-204 (2013).

124. Hertiani T, Pratiwi SU. Hydnophytum formicarum Jack ethanol extract modulates quorum sensing-controlled pathogenicity in Pseudomonas aeruginosa. *Pak J Pharm Sci.* 28(5), 1691-1697 (2015).

125. Jayasinghe UL, Jayasooriya CP, Bandara BM, Ekanayake SP, Merlini L, Assante G. Antimicrobial activity of some Sri Lankan Rubiaceae and Meliaceae. *Fitoterapia* 73(5), 424-427 (2002).

126. Wongsariya K, Phanthong P, Bunyapraphatsara N, Srisukh V, Chomnawang MT. Synergistic interaction and mode of action of Citrus hystrix essential oil against bacteria causing periodontal diseases. *Pharm Biol* 52(3), 273-280 (2014).

127. Kooltheat N, Kamuthachad L, Anthapanya M *et al.* Kaffir lime leaves extract inhibits biofilm formation by Streptococcus mutans. *Nutrition* 32(4), 486-490 (2016).

128. Al-Sohaibani S, Murugan K. Anti-biofilm activity of Salvadora persica on cariogenic isolates of Streptococcus mutans: in vitro and molecular docking studies. *Biofouling* 28(1), 29-38 (2012).

129. Sofrata A, Santangelo EM, Azeem M, Borg-Karlson AK, Gustafsson A, Putsep K. Benzyl isothiocyanate, a major component from the roots of Salvadora persica is highly active against Gram-negative bacteria. *PLOS ONE* 6(8), e23045 (2011).

130. de Souza RC, da Costa MM, Baldisserotto B *et al.* Antimicrobial and synergistic activity of essential oils of Aloysia triphylla and Lippia alba against Aeromonas spp. *Microb Pathog* 113, 29-33 (2017).

131. Ghosh S, Indukuri K, Bondalapati S, Saikia AK, Rangan L. Unveiling the mode of action of antibacterial labdane diterpenes from Alpinia nigra (Gaertn.) B. L. Burtt seeds. *Eur J Med Chem.* 66, 101-105 (2013).

132. Rao K, Ch B, Narasu LM, Giri A. Antibacterial activity of Alpinia galanga (L) Willd crude extracts. *Appl Biochem Biotechnol.* 162(3), 871-884 (2010).

133. Diao WR, Zhang LL, Feng SS, Xu JG. Chemical composition, antibacterial activity, and mechanism of action of the essential oil from Amomum kravanh. *J. Food Prot.* 77(10), 1740-1746 (2014).

134. Packiavathy IA, Priya S, Pandian SK, Ravi AV. Inhibition of biofilm development of uropathogens by curcumin - an anti-quorum sensing agent from Curcuma longa. *Food Chem.* 148, 453-460 (2014).

135. Gupta A, Mahajan S, Sharma R. Evaluation of antimicrobial activity of Curcuma longa rhizome extract against Staphylococcus aureus. *Biotechnol Rep* 6, 51-55 (2015).

136. Chan EWC, Lim YY, Omar M. Antioxidant and antibacterial activity of leaves of Etlingera species (Zingiberaceae) in Peninsular Malaysia. *Food Chem.* 104(4), 1586-1593 (2007).

137. Kabir SR, Hossen A, Zubair A *et al.* A new lectin from the tuberous rhizome of Kaempferia rotunda: isolation, characterization, antibacterial and antiproliferative activities. *Protein Pept Lett* 18(11), 1140-1149 (2011).

138. Ghasemzadeh A, Jaafar HZ, Ashkani S *et al.* Variation in secondary metabolite production as well as antioxidant and antibacterial activities of Zingiber zerumbet (L.) at different stages of growth. *BMC Complement Altern Med* 16, 104 (2016).

139. Kumar R, Jandaik S, Patial P. Screening of medicinal plants of Himachal Pradesh for efflux pump inhibitory activity against Escherichia coli. *J Pharmacogn Phytochem.* 5(3), 96-100 (2016).

140. Al-Bayati FA, Al-Mola HF. Antibacterial and antifungal activities of different parts of Tribulus terrestris L. growing in Iraq. *J. Zhejiang Univ. Sci. B* 9(2), 154-159 (2008).
